# Simplified equations for object trajectories in rotating space habitats and space juggling

**DOI:** 10.1038/s41526-023-00328-6

**Published:** 2023-10-12

**Authors:** R. Adam Dipert

**Affiliations:** Department of Physics, North Carolina State University, 2401 Stinson Drive, Box 8202, Raleigh, 27695 NC USA

**Keywords:** Applied physics, Applied mathematics

## Abstract

Rotating space habitats provide artificial gravity as a physiological countermeasure for long-term space habitation, though the lived experience of a person in these habitats requires further investigation. Movement planning will require adaptation to the Coriolis and centrifugal forces. The multicultural arts of juggling may offer potential psycho-physiological countermeasures for some individuals and provide interesting insights into movement planning and arts in both microgravity and rotating habitats. Previously developed equations of motion for thrown objects in rotating habitats have not been centered within the lived rotating environment. Here, I show a set of simplified equations for object trajectories in rotating environments and their underlying mathematical framework. The full set of possible trajectories for objects thrown in rotating environments is provided and a simplified approach to the Coriolis and centrifugal force differential equation using complex algebra is demonstrated. Experimentation reported in this article was conducted on parabolic flights and an analog weightlessness and rotating apparatus. Near the surface of the Earth, thrown objects travel along parabolas. In rotating space environments, thrown objects will travel along a set of mathematical curves known as roulettes, created by a fixed circle and rolling line with generator point connected to the line. These roulette trajectories will be the everyday experience of every person living in a rotating space habitat, always.

## Introduction

It is well understood that psycho-physiological countermeasures are necessary for long-duration space flight to ensure space travelers’ health^1–6^. Rotating space habitats are capable of providing artificial gravity with existing technology for extended periods. Movements, object motion, and the vestibular experience in rotating environments are influenced by the Coriolis and centrifugal forces in meaningful ways^1,7–9^. These forces have a direct influence on a person’s ability to anticipate the results of physical interactions with objects and the environment^10^.

During the research reported here, while attempting to develop a useful set of expectations for anticipating thrown object motion in a rotating reference frame, a number of mathematical and physiological models were used. Common mathematical approaches to the equations of motion in rotating reference frames use linear algebra and are oriented from an external, inertial reference frame^7^. Please see the Supplementary videos for examples. Unfortunately, approaching the equations in this manner results in mathematics that is outside the lived experience in a rotating habitat and involves daunting mathematics.

Thrown or dropped objects close to the surface of planets follow parabolic trajectories due to gravity. Seven month old children already begin to understand that there is a direction of gravity that affects object motion^11^, while adults are able to take this trajectory into account intuitively^12^. In rotating space habitats, the trajectory of thrown or dropped objects will be different, but it is not clear what mathematical structure these trajectories will follow. While T.W. Hall noticed that the trajectory of a “dropped ball in a rotating space habitat follows the path of an involute”^7^, I sought to further explore this phenomenon and determine whether that is the entire story.

This work began as a mission to develop a technique for juggling in microgravity, and resulted in observations about life in rotating space habitats gained through embodied practice. Humanity has at least a 4000 year history of engaging in the cultural arts of object manipulation for entertainment (juggling)^13^. Even though juggling has been practiced in space on a Space Shuttle by Donald Williams during STS-51-D mission^14^ and on the ISS by Richard Garriott and Greg Chamitoff during Soyuz TMA-13/TMA-12 missions^15^, those approaches were oriented from an Earth-biased concept of juggling^16^.

During this investigation, the first observation was that the maximum moment of inertia eigenvector of the human body in the extended supine position (see Figs. 4, 5) in microgravity is oriented along the anterior-posterior (AP) axis^17,18^. This means that the most stable way a human body can rotate in microgravity while in the extended supine position is in a “cartwheel” motion. The second observation was that the center of masses of thrown objects move in straight lines in microgravity. Combining these concepts, we find that a person can rotate about the AP axis and throw balls “down” to themselves to catch at a later time when the body is in a different orientation.

Parabolic flights were used to explore body rotation and an apparatus was constructed to investigate the combination of body and object movement. After practicing and filming this, I noticed that in the rotating frame, the balls appear not to move in lines, but instead along curves. The curves are mentioned in many places in literature and on the internet but no evidence of their fundamental mathematical structure could be found^7,19,20^. Thus, the study sought to investigate whether there is a single mathematical object that describes all the trajectories of thrown objects in a rotating space habitat.

The Results section presents the mathematical findings of this study, followed by the results of physical experimentation with object motion in a rotating reference frame. In the Methods section, I describe the experimental techniques used and provide more detailed descriptions of the development of the equations, as well as a comparison between complex and linear algebraic approaches to the equations.

## Results

### Overview

Four useful ways to approach the mathematics associated with the trajectories of thrown objects in weightlessness observed in a rotating reference frame were found during this investigation. These perspectives are:

1. Exploration of the equations that describe the trajectories.
2. Visual representation of the trajectories in both inertial and rotating reference frames.
3. Examination of the underlying mathematics of roulettes, generated by a fixed circle and rotating line, and their relation to the equations.
4. Demonstrating that the equations take into account the Coriolis and centrifugal forces.

During the investigation of juggling techniques, I employed the mathematical perspectives outlined above. The study included significant focus on the practical aspects of utilizing these mathematical perspectives rather than solely on their development. At the end of this section, I also discuss the results of experimentation with the trajectories.

The reader will also find videos in the Supplementary materials associated with this document online. The videos are films and animations showing the creation of the roulette, recordings and tracking of data, and animations of rotating space habitats.

### Standard equations

Let’s begin to understand and develop the equations of motion using the most straightforward method. Any thrown object in microgravity does not experience a force and, therefore, has a constant velocity, which means it travels along a line. Parametric equations of lines along each axis in a plane can be written as

1 $$X(t)=\dot{X}\,t+{X}_{0}$$

2 $$Y(t)=\dot{Y}\,t+{Y}_{0}$$

where *t* is time, the subscript 0 indicates the initial position, and the dot represents the time derivative. We can then map these equations onto the complex plane by writing the trajectory in an inertial reference frame.

3 $${T}_{inertial}(t)=X(t)+i\,Y(t)$$

We can now manipulate the equation with a rotation to have the trajectories in the rotating reference frame.

4 $$\begin{array}{lll}{T}_{rotating}(t)=\,{e}^{-i\omega t}\times {T}_{inertial}(t)\\\qquad\qquad\;\; =\,{e}^{-i\omega t}\,\left(X(t)+i\,Y(t)\right)\end{array}$$

where *ω* is the angular velocity of the system and *i* is the complex unit. This form of the equations is useful, as it helps to establish a clear connection between the inertial and rotating reference frames. I will call it a standard form and referred to as the “parameterized equation.” For the rest of this paper, all equations will be in the rotating frame, therefore, the *rotating* subscript will be dropped.

In Eq. (4), the argument is negative. This means that the rotation applied to the linear trajectory is clockwise which is opposite of the rotation of the reference frame. Returning to a Cartesian coordinate system momentarily, we would naturally equate the positive real direction to the positive x direction and the positive imaginary direction to the positive y direction. Let it be a right-handed coordinate system. This aligns the coordinate system rotation vector *ω* with the positive z-axis.

We can consider another standard form which separates the time-dependent and constant components of the linear part of Eq. (4). First substitute Eqs. (1), (2) into (4), do some sorting, and we have the following:

5 $$T(t)={e}^{-i\omega t}\,(at+b)$$

where $a,\,b\in {{C}}$. This equation allows an intuitive starting point, namely *T*(*t* = 0) = *b*. One may be inclined to try to interpret *a* as the velocity at time zero but $\dot{T}(t=0)=a-i\,b\,\omega$. Nonetheless, the initial position and velocity can easily be extracted from the equation, and we can safely differentiate it from the other equations by this quality, thus, let’s call it the “point-velocity equation”.

A particularly big problem with both the parameterized (Eq. (4)) and point-velocity (Eq. (5)) equations is that they give preference to the inertial frame by having their linear components so explicitly expressed. For those living or working in a rotating reference frame, one would prefer to use equations represented in that frame. Therefore, this final equation, which we might call the “point-point equation,” might be the most useful. When practicing throwing balls in the apparatus described in Analog Microgravity Environment, I found this equation to be the most consistent with my lived experience and objectives.

6 $$T(t)={e}^{-i\omega t}\left[\frac{t}{\tau }\left({e}^{i\omega \tau }\,{\Omega }_{2}-{\Omega }_{1}\right)+{\Omega }_{1}\right]$$

where ${\Omega }_{1},\,{\Omega }_{2}\,\in {{C}}$ are the starting and ending positions, respectively, as observed within the rotating frame, *τ* is the amount of time between the release and catch, *ω* is the angular velocity of the system, and t is time. It is worth noting that Ω_1_ and Ω_2_ can easily be represented in rectilinear (*c* + *di*) or angular (*re*^*iψ*^) coordinates, where *r* is the distance from the axis of rotation and *ψ* is the angle from the preferred real axis orientation. When comparing Eq. (6) to Eq. (5), it may be noted that the substitutions *a* = (1/*τ*)(*e*^*iωτ*^ Ω_2_ − Ω_1_) and *b* = Ω_1_ prove their equality. A derivation of Eq. (6) can be found in the Derivation of Point-Point Equation section.

### Observable trajectories

**Fig. 1:**
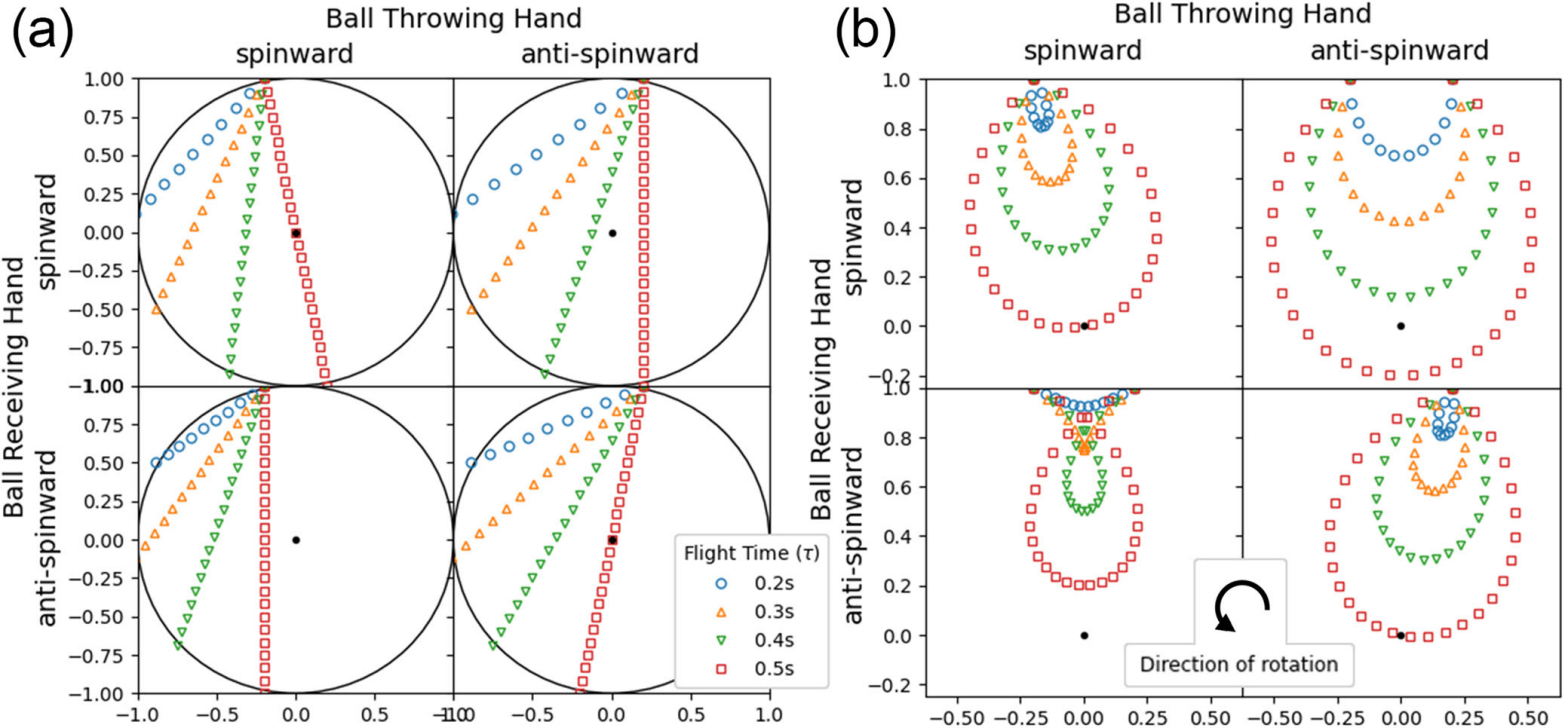
Trajectories of thrown objects in non-rotating and rotating environments (in weightlessness). Trajectories starting from the *spinward* and *anti-spinward* directions are shown in the left and right columns, respectively. Trajectories ending in the *spinward* and *anti-spinward* directions are shown in the top and bottom rows, respectively. **a** Trajectories in inertial (non-rotating) frame. **b** Trajectories in rotating frame. Using equation (6) with $| {\Omega }_{1}| =| {\Omega }_{2}| =\sqrt{{1}^{2}+0.{2}^{2}}\,m$, *ω* = 2*π* *rad*/*s*, and 0.2 *s* < *τ* < 0.5 *s* in increments of 0.1 *s*, and rotated by *i* for reference. Each point on each plot is spaced by 17 *ms*. In the case of Space Juggling, the left column of the plots are throws made from the left hand. The right column are throws from the right hand. The first and second rows are throws caught with the left and right hands, respectively.

Some of the trajectories one may observe in both the inertial and rotating frames are plotted in Fig. 1. The plots are given for a number of throwing and catching positions and as well as different lengths of time between the throw and catch.

The rotation is stable in these systems and oriented counterclockwise from the viewer’s perspective. The *spinward* direction is the direction in which the habitat is rotating, in this case counterclockwise. The *antispinward* direction is opposite the direction of rotation, in this case clockwise. The four panels of each figure show trajectories starting from the spinward direction in the left column and trajectories starting from the antispinward direction in the right column. Trajectories ending on the spinward and antispinward directions are shown in the top and bottom rows, respectively. Thus, the upper left panel of each figure is a spinward to spinward throw, the top right panel of each figure is an anti-spinward to spinward throw, etc.

Figure 1a shows a set of trajectories as seen from an inertial reference frame. You will notice that the trajectories are straight lines forming chords within the circle. These plots can be created by removing the leading rotating exponential term from Eq. (6).

The same trajectories are shown again in Fig. 1b but instead are shown in the rotating reference frame. Notice the symmetry in the patterns, especially the similarities between the top left and bottom right panels. Some of the most interesting curves are found in the lower left panel. Here, we discover a full loop crossing itself as well as a cusp (shown in dashed orange). As described in the Roulettes section, the cusp is actually an involute, while the curves crossing the black dot (center of circle and axis of rotation) are Archimedean spirals.

### Roulettes

**Fig. 2:**
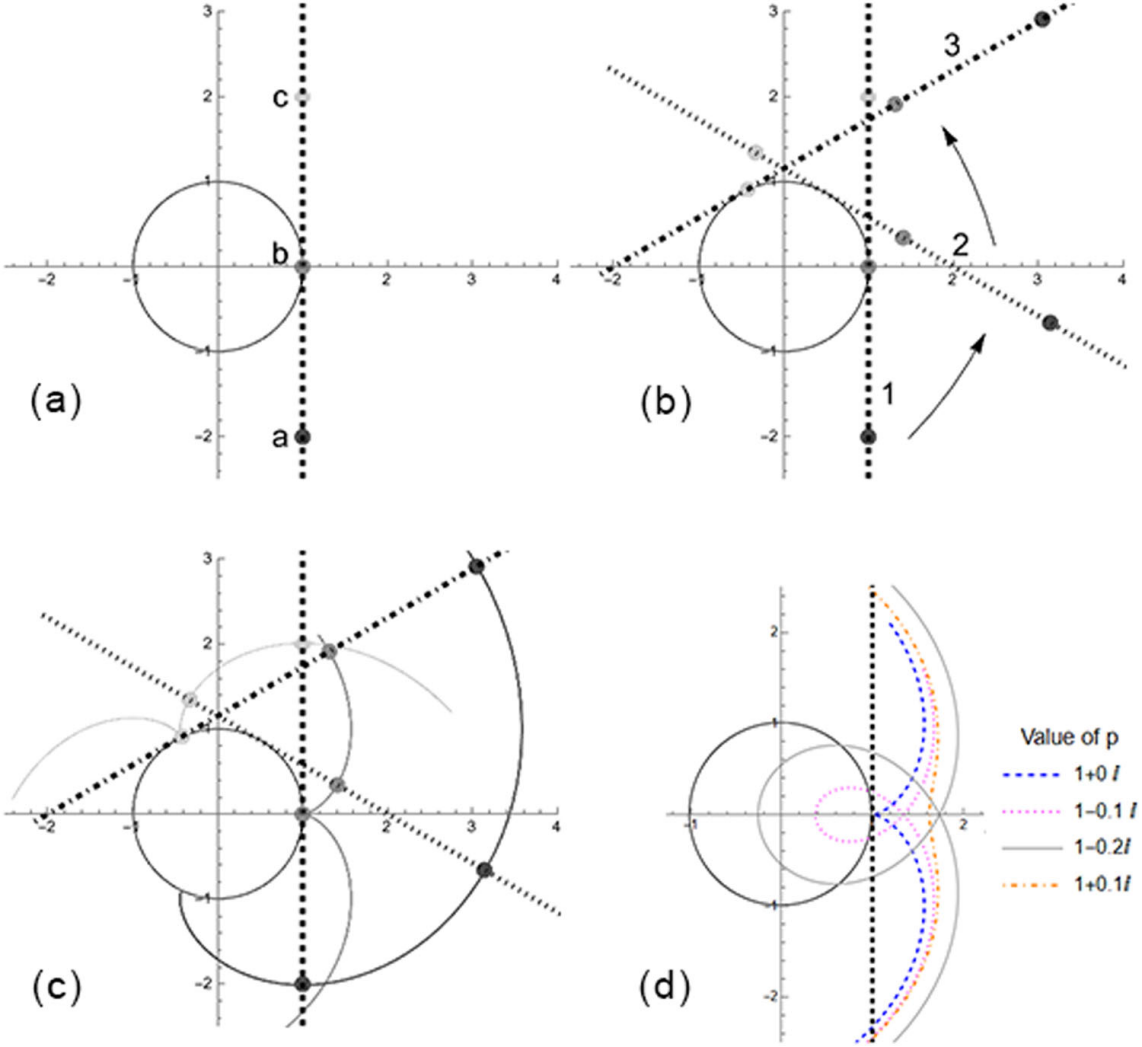
Roulette geometry of involute and other patterns. The technique for creating the roulette curves with a fixed circle, rolling line, and generator point positioned on the line and the result of offsetting the generator from the line which reconstructs the general set of curves. **a** Begin with a line adjacent to a circle. Note that three points on the line have been marked: *a*, *b*, and *c* with decreasing opacity. **b** Now roll the line around the circle without slipping. Here the line is rolled counterclockwise. Note the locations of the points *a*, *b*, and *c* on the line as it rolls at times *1*, *2*, and *3*. **c** Tracking the trajectories of the points on the lines, the roulette curves described by Eq. (6) are found. In this case, the generator point is located on the line, and the involute of a circle is created. **d** Adjusting the value of *p* in Eq. (7), we recover all of the trajectories shown in Fig. 1b but in this case generated by the roulette construction. The Roulettes subsection contains important caveats.

The trajectory of a ball which is “dropped” while in a rotating space habitat follows the path of an involute, as T.W. Hall has noted^7^. “An involute of a curve is the locus of a point on a piece of taut string as the string is either unwrapped from or wrapped around the curve”^21^. The involute can also be created by following a point on a line as the line is rolled without slipping along the outside of a circle. Figure 2a–c demonstrate this construction of an involute.

The equation of an Archimedean spiral using linear algebra is easily written as *x* = (*vt* + *c*)*cos*(*ωt*) and *y* = (*vt* + *c*)*sin*(*ωt*), thus equation (5) clearly becomes an Archimedean spiral when $a,\,b\in {{R}}$ instead of being complex numbers. With $a,\,b\in {{C}}$, the curve simply must cross the axis of rotation to be an Archimedean spiral. Interestingly, an Archimedean spiral can be created by rolling a line without slipping around a fixed circle, and following a point (called a “generator”) which is offset by the radius of the circle in the direction of the circle.

The operations described above to create involutes and Archimedean spirals by rolling lines around circles are types of objects mathematically called “roulettes.” A roulette is created by taking a fixed (*f*) and rolling (*r*) curves and rolling *r* along *f* without slipping, while tracking a point *p* (called the “generator”) which is attached to the rolling curve^22^. In the equations below, ${f}^{{\prime} }(t)$ and ${r}^{{\prime} }(t)$ are the derivatives of the fixed and rolling curves, respectively. The generator *p* can have an offset from *r*. If we take $f,\,r,\,p\in {{C}}$, the following formula can be used to create a roulette.

7 $$f(t)+(p-r(t))\frac{{f}^{{\prime} }(t)}{{r}^{{\prime} }(t)}$$

Involutes are a type of roulette where *f*(*t*) is a circle, *r*(*t*) is a line, and *p* is a point on *r*(*t*) and moves with *r*(*t*). Archimedean spirals are a type of roulette where *f*(*t*) is a circle, *r*(*t*) is a line, and *p* is offset from *r*(*t*) by *R* in the direction of the center of the circle *f*(*t*).

All the curves described by the standard equations above are roulettes. A mapping between the point-velocity form (Eq. (5)) and roulette equation (Eq. (7)) can be written if we assume the fixed curve is a circle with radius *R* while the rolling curve is a line.

8 $$f(t)=\,R\,{e}^{-i\omega t}$$

9 $$r(t)=\,t$$

We plug these ansatz into Eq. (7) and equate to Eq. (5) to determine the mapping.

10 $$\begin{array}{ll}f(t)+(p-r(t))\frac{{f}^{{\prime} }(t)}{{r}^{{\prime} }(t)}=\,R{e}^{-i\omega t}+(p-t)\left(-i\omega R\,{e}^{-i\omega t}\right)\\\qquad\qquad\qquad\qquad\quad =\,{e}^{-i\omega t}\,\left(i\omega Rt+R(1-i\omega p)\right)\\\qquad\qquad\qquad\qquad\quad =\,{e}^{-i\omega t}\,(at+b)\end{array}$$

Thus, the mapping between the roulette and “point-velocity” equations is

11 $$R=\frac{a}{i\,\omega }$$

12 $$p=\frac{1}{i\,\omega }-\frac{b}{a}$$

Given any curve from a standard equation, we can identify the radius *R* of the fixed circle and orthogonal offset *p* of the generating point. Q.E.D. Figure 2d shows a number of the curves found in Fig. 1 using the roulette construction.

Note that in Eq. (11), $R\in {{{\mathcal{C}}}}$, and can be written in polar form separating the magnitude ∣*R*∣, which is the radius of the fixed circle and a phase, which uselessly rotates the circle.

Care must be taken to understand the relationship between *r*(*t*) and *p* because the effect of *p* having real and imaginary components is not obvious. Eq. (9) defines *r*(*t*) as a real-valued function. If plotted alone on the imaginary plane, *r*(*t*) will only exist on the horizontal real axis. Eq. (7) shows the relationship between *p* and *r*(*t*) within the parentheses as $\left(p-r(t)\right)$. In this format, it is clear that an imaginary component of *p* will provide a vertical offset of the generator, while a real component will provide a horizontal offset.

This component $\left(p-r(t)\right)$ of Eq. (7) is then multiplied by $\frac{{f}^{{\prime} }(t)}{{r}^{{\prime} }(t)}$, where ${r}^{{\prime} }(t)$ is always a constant, because r(t) is a line. Note this means that ${r}^{{\prime} }(t)\,\ne\, 0$ for any *t*, so there should be no concern about ${r}^{{\prime} }(t)$ being in the denominator and possibly creating an undefined value. Thus, we have $(p-r(t))({f}^{{\prime} }(t))$. From Eq. (8), we see that *f*(*t*) is a circle. Multiplying its derivative ${f}^{{\prime} }(t)$ by another function performs a rotation on that function. The result of these features is that the imaginary and real components of *p* follow the line of *r*(*t*) as it rolls around *f*(*t*), where imaginary components of *p* will result in perpendicular offsets, and real components will result in values sliding along the direction of *r*(*t*).

### Coriolis and centrifugal forces

As we know, the curves which have been discussed occur not from actual forces, but as a result of having a particular perspective in a rotating reference frame. Perceived accelerations can be explained by the fictitious Coriolis and centrifugal forces, which are most often written in the form of the following differential equation.

13 $$m{{{{{a}}}}}^{{\prime} }=-2m{{{{\omega }}}}\times {{{{{v}}}}}^{{\prime} }-m{{{{\omega }}}}\times ({{{{\omega }}}}\times {{{{{r}}}}}^{{\prime} })$$

where ${{{{{a}}}}}^{{\prime} }$, ${{{{{v}}}}}^{{\prime} }$, ${{{{{r}}}}}^{{\prime} }$ are the acceleration, velocity, and position, respectively, in the rotating frame, *ω* is the angular velocity vector, and *m* is the mass of the object. In Eq. (13), the first term on the right side of the equal sign is the Coriolis force and the second term is the centrifugal force.

Make note that each term has a cross product with the *ω* vector. This tells us that the forces only act in the plane perpendicular to the axis of rotation. Although in the most general cases, the velocity vector of a thrown object may have a component parallel to *ω*, we know that velocity component is always linear and experiences no fictitious forces. For this reason, we will restrict the conversation here to deal solely with motion in the plane perpendicular to *ω*.

The cross product gives a resultant vector perpendicular to the two vectors it is acting on. The cross product acts on the angular velocity *ω* and a general vector in the plane *u*. Using complex algebra, the cross product operation (*ω* × *u*) is homomorphic with the multiplicative product *iωu*, where *ω* = ∣*ω*∣ and $u\in {{C}}$. This complex product gives the same result as the cross product in the plane.

We will proceed to solve Eq. (13) using Eq. (5) as our ansatz solution. Below are the first and second time derivatives.

14 $$\begin{array}{ll}T=\,{e}^{-i\omega t}(at+b)\\ \dot{T}=\,{e}^{-i\omega t}[(-i\omega )(at+b)+a]\\ \ddot{T}=\,-{e}^{-i\omega t}[2i\omega a+{\omega }^{2}(at+b)]\end{array}$$

All terms in Eq. (13) are multiplied by the scalar *m* and thus we can remove it. Rewriting Eq. (13) in terms of *T*, rewriting using complex algebra, and inserting the time derivatives, we find.

15 $$\begin{array}{ll}\ddot{T}=\,-2{{{{\omega }}}}\times \dot{T}-{{{{\omega }}}}\times ({{{{\omega }}}}\times T)\\\quad =\,-2(i\omega )\dot{T}-(i\omega )(i\omega )T\\\quad =\,-{e}^{-i\omega t}[2i\omega a+{\omega }^{2}(at+b)]\end{array}$$

Q.E.D.

Of course, this result is no surprise. The contributions in this paper made to solving this differential equation (Eq. (13)) are (1) the simplicity of the solutions (by using complex algebra) and (2) showing the solution can be generated by roulettes.

### Experimental results

**Fig. 3:**
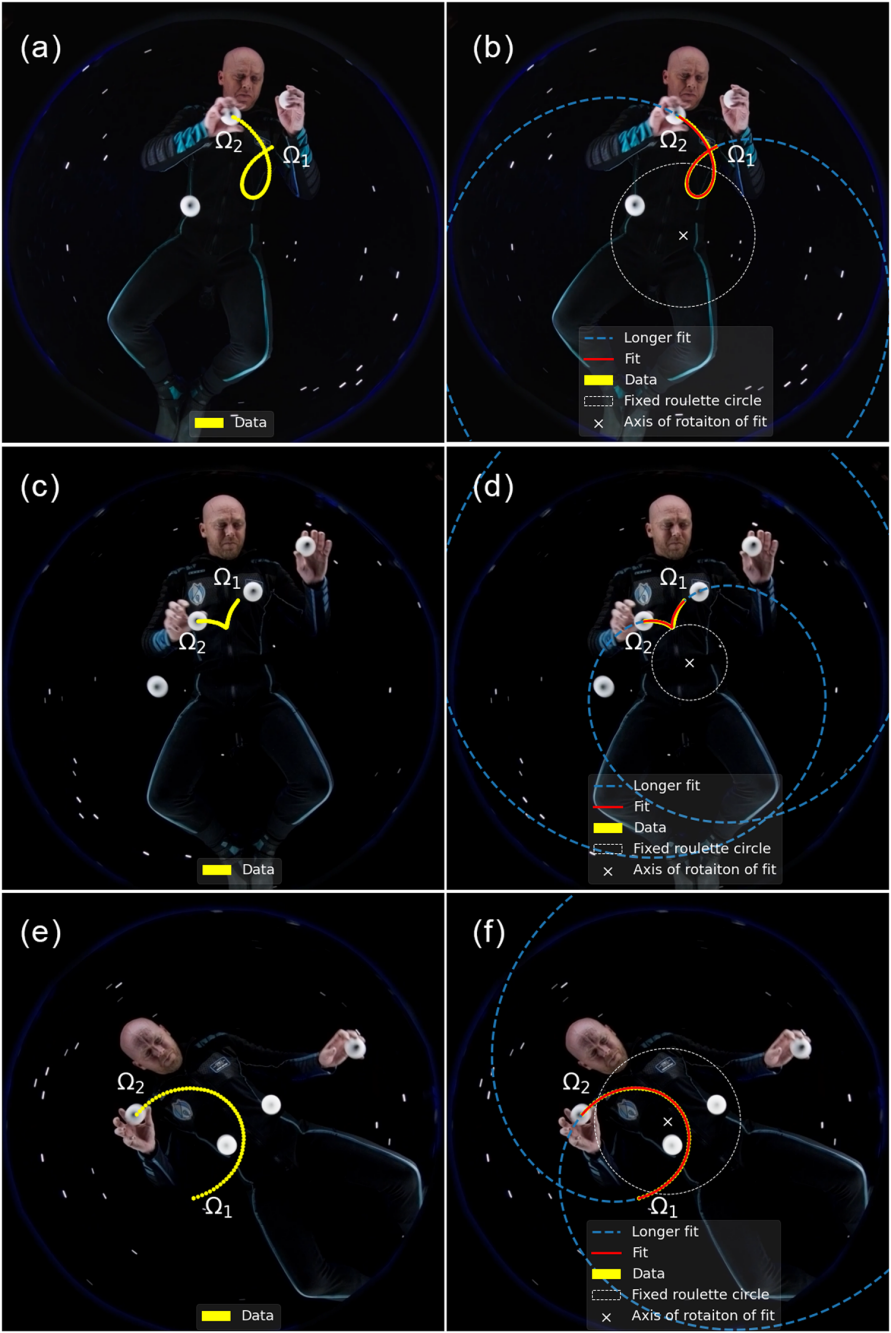
Observed trajectories and roulette visualization. A variety of trajectories recorded in the rotating reference frame using the apparatus described in the Analog Microgravity Environment section and fit using Eq. (6). See text in the Experimental Results for details. **a** This trajectory can be found in the lower left panel of Fig. 1b. **b** Shows *f*(*t*) of roulette, fit, and extrapolation of curve from Fig. 3a. **c** This trajectory can be found in the lower left panel of Fig. 1b and is close to being an involute. **d** Shows *f*(*t*) of roulette, fit, and extrapolation of curve from Fig. 3c. **e** This trajectory can be found in the upper left panel of Fig. 1b. **f** Shows *f*(*t*) of roulette, fit, and extrapolation of curve from Fig. 3e. Written informed consent was provide by the subject for the use of this image.

Using an analog environment (described in the Analog Microgravity Environment section), trajectories very close to those shown in Fig. 1b were recorded. Figure 3 shows a series of trajectories which were recorded and then tracked. Films of these trajectories being generated in the rotating frame can be found in a YouTube video^23^ and more general videos of larger sets of trajectories can be found through that same user account.

The left column of Fig. 3 shows the data points tracked and connected, as well as the initial position of releasing the ball Ω_1_ and the final point before catching the ball Ω_2_.

Recordings were made at 60 frames per second giving time resolution of 16.7 *ms* and with video resolution of 1920 by 1080 pixels. The parameters of Eqs. (5), (6), and (7) were fit to the data. The mean *d* and standard deviation *σ*_*d*_ between the location of the data pixel and calculated fit pixel are found at the bottom of Table 1.

**Table 1:** Fit parameters for equations (6) to (10) found for thecurves shown in figures 3.

Fit Values For Figures			
Figure Numbers	3a and 3b	3c and 3d	3e and 3f
a	32.9 − 375.8 i	113.7 − 111.2 i	212.8 + 338.2 i
b	81.2 + 215.7 i	−15.6 + 147.4 i	−69.3 − 187.2 i
Ω_1_	81.2 + 215.7i	−15.1 + 147.4i	−69.3 − 187.2i
Ω_2_	−8.1 + 287.7 i	−109.9 + 99.7i	−207.6 + 16.9i
*τ* (s)	1.27	1.43	1.00
*R*	174.4 + 15.3 i	64.1 + 65.5 i	−150.4 + 94.6 i
*p*	0.551 + 0.200 i	0.716 − 0.020 i	0.489 + 0.547 i
*ω* (rad s^−1^)	−2.15	−1.73	−2.25
*d* ± *σ*d	$0.1338\hat{{{{\rm{A}}}}}\pm 0.067$	$0.2127\hat{{{{\rm{A}}}}}\pm 0.1364$	$0.0327\hat{{{{\rm{A}}}}}\pm 0.0354$
*n*	76	86	60

The last line gives the mean displacement and standard deviation between the data collected and fit analysis. All terms without explicitly given units have units of length, “pixels.” The number of pointsn used for each fit are shown.

To simulate weightless conditions, balls were rolled on a taut clear vinyl surface rather than floating through free air. A camera was placed under the surface facing up. The camera was attached to a motor which provided rotation, generating a rotating reference frame. Due to this set up, the balls were subject to friction, attractive forces (as the surface distorted due to the mass of the balls), and to a small degree the Euler force (due to the camera rotational velocity being controlled by hand and not always rotating at constant velocity). Even though these forces had an impact on the trajectories of the balls in general, patterns were selected for analysis in this paper that showed minimal distortion from interaction with the surface. The observation that the balls moved in fairly straight lines is quantified in the mean displacement and standard deviations found in Table 1.

## Discussion

Much thought has been given to the experience of the lives of people who will live in rotating space habitats and a number of experiments have been conducted^24–26^. In previous literature, the trajectories described in this paper have been identified in simulation or using brute force methods^7,19^. While these approaches showed the trajectories described, they were not represented in a manner conducive to a life lived in a rotating space habitat. This is no surprise. People had not started to evaluate the reality of the human body in microgravity until the development of the space age. There is no reason for them to have conceived of the microgravity framework previously. We are nearing the moment when rotating space habitats may become a realistic environment for habitation, and very few people have lived and experienced problem solving in rotating environments for extended periods.

The words of astronaut Gerald Carr, who spent 84 days in space, most clearly expresses the contemporary perspective on this topic. “The humans on the rim of a rotating space station that develops artificial gravity have to see the velocity as a linear rather than curvilinear orbit, or it doesn’t work because of Coriolis acceleration on the inner ear”^27^.

The insights described in this paper developed after spending about 500 h practicing object manipulation in the rotating analog environment described above. Initially, I attempted to plan the object trajectories in an inertial reference frame, as previous literature and mathematics would suggest one does. This caused headaches. As soon as I started to think in terms of the roulette curves, I had more accurate motion planning and the headaches went away.

The overwhelming majority of papers published about object trajectories in rotating reference frames are expressed using linear algebra oriented within an inertial frame. One of the intentions of this paper is to outline a simpler approach using complex algebra. Taking derivatives and solving the differential equations has been shown to be exceedingly manageable and the reader is invited to investigate the appendices for further insight. Although the equations of motion are most simply understood by the planet-bound mind by writing the equation in the inertial frame and rotating it, this may not be as useful when one inhabits a rotating reference frame.

The concepts expressed in this paper are the infrastructure needed to plan all non-accelerating (in the inertial frame) trajectory tasks in rotating space habitats with constant angular velocity when air resistance does not significantly influence the movement of the object. Using this as a framework, I have developed the art form of Space Juggling which is only the beginning of what is to come when entertainment and sports are sincerely brought to space^28^. Any circus connoisseur will easily have insight about the vast number of ways the trajectories of the centers of mass of objects are used in contemporary circus and how those seeds might expand into a true space circus experience. We can hope to live in a more richly enlivened solar system where that level of creativity is combined with altered gravitational environments to manipulate trajectories that are, as of yet, only theorized.

## Methods

### Ethical review board

These studies were ethically reviewed by the institutional review board at North Carolina State University in Raleigh, NC (protocol number 25523), and were deemed to be in accordance with Common Rule 45 CFR 46 and the Declaration of Helsinki. The sole subject of the video recordings is the PI (R. Adam Dipert) demonstrating the rotation technique on a parabolic flight and in an aerial apparatus. Written informed consent was obtained from the subject.

### Physical phenomena relevant to this work

The two primary phenomena central to this work are: (1) the body rotating about the AP axis (similar to a “cartwheel”), and (2) balls moving in straight lines in an inertial reference frame. The latter can easily be found in many videos online^29^. The first part of this section deals primarily with establishing the body’s rotation, techniques employed to manifest this rotation, and observations in a rotating reference frame.

Like all three dimensional objects, the human body has three principal axes which are eigenvectors of the moment of inertia tensor. When the body’s angular momentum is aligned with the maximum axis, the most stable rotation occurs. Computation reveals that the maximum axis of the human body in a bent-knee position (Fig. 5a) and extended supine position (see Fig. 1b of ref. ^18^) are aligned with the AP axis. Interestingly, this is the only way a torque-free human body can rotate while always facing the same direction.

### Parabolic flights

**Fig. 4:**
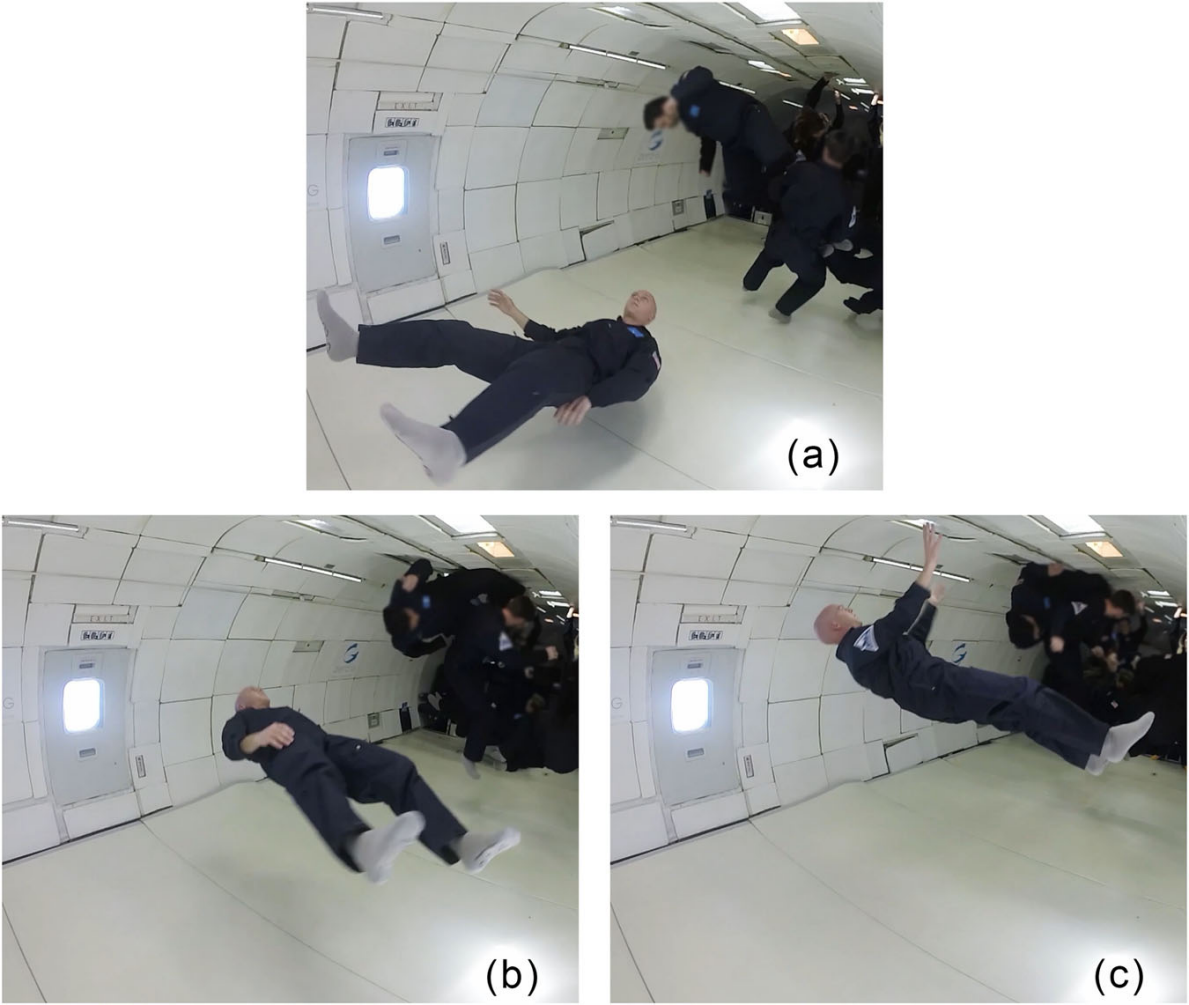
Parabolic flight video frames. Rotation about anterior-posterior axis while in extended supine position on parabolic flight. Recorded Mar 3, 2018. Note that linear drift toward to ceiling of the plane results in the body moving up in the frame throughout the sequence. **a** Beginning of maneuver. **b** Middle of maneuver. **c** End of maneuver (90^∘^ rotation about AP axis). Written informed consent was provide by the subject for the use of this image.

The rotation of the body was tested on a commercial parabolic flight in 2018. Figure 4 shows a sequence of images taken from video where the body is rotating stably for a half rotation in the cartwheel. There are many videos online showing astronauts doing forward flips and spins about the minimum principal axis, unfortunately, none could be found showing astronauts doing the type of cartwheel motion previously mentioned. Although, the mathematical dynamics of the human body within torque-free environments are well understood, these tests directly demonstrate the concept.

### Analog microgravity environment

**Fig. 5:**
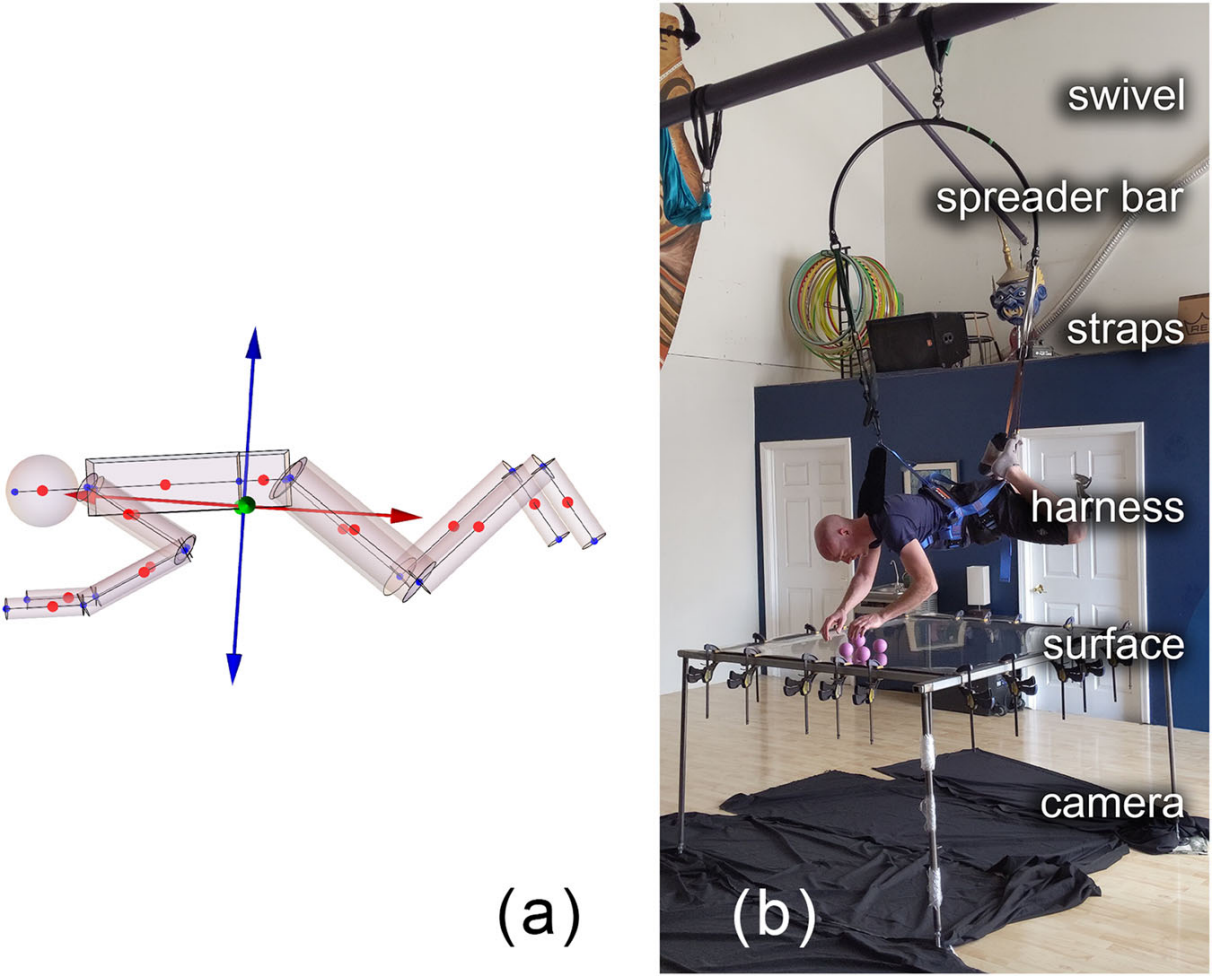
Human body model and orientation in analog apparatus. Simulation of human body in desired position for Space Juggling technique, and position and orientation of the human body in the analog apparatus. **a** Model of articulated human body with moment of inertia eigenvectors shown and oriented outward from the whole body’s center of mass. The blue and red arrows are the maximum and minimum eigenvectors, respectively. **b** Microgravity analog apparatus allowing for balls to travel along straight lines while body rotates around maximum moment of inertia axis. Written informed consent was provide by the subject for the use of this image.

Access to parabolic flights is often very expensive and microgravity parabolas are short lived, lasting 20–30 s each. Therefore, a ground based analog apparatus was constructed to allow affordable and longer duration access to an experimental environment which simulated relevant physics. In the analog, the body was suspended in a vest-harness facing down. The harness was attached to a swivel which allowed rotation about the AP axis (see Fig. 5b). This allowed for tens of minutes of practice rather than tens of seconds.

Linear motion of the balls was accomplished by rolling them on a clear vinyl sheet pulled taut with hooks and grommets, and oriented horizontally. The balls were hollow “stage balls” made of PVC, having diameter 62 mm, and mass 75 g. A camera was placed under the vinyl sheet facing up to capture the image from the front of the body. The camera was then rotated using a motor. The trajectories discussed in the Results section were then realized.

### Derivation of point-point equation

Euler’s Formula expresses a rotating unit circle in the complex plane, *e*^*ix*^ = *cos*(*x*) + *i* *sin*(*x*). Using this, we can formulate the equation of a point rotating around the origin at a distance *A*.

16 $${T}_{A}(t,{\omega }_{0})=A\,{e}^{i{\omega }_{0}t}$$

where *i* is the imaginary unit, *ω*_0_ is the rotational velocity, and *t* is time. In this construction, as *t* increases, the point rotates counterclockwise. In the case of a rotating habitat, Eq. (16) would be some fixed point in the rotating reference frame, likely on the inner surface of the ring upon which people would stand and equipment would rest. In the case of Space Juggling, this would likely be the orientation of the spine.

We can extend a line from *T*_*A*_ out in each perpendicular direction by length *S* by starting with a similar complex exponential base and rotation using *i* and −*i*. For Space Juggling, these are the locations of the hands. These locations are *spinward* and *anti-spinward* of *T*_*A*_(*t*, *ω*_0_). (See the Observable Trajectories section for more details about this terminology).

17 $$\begin{array}{ll}{p}_{S}(t,{\omega }_{0})=A\,{e}^{i{\omega }_{0}t}+iS\,{e}^{i{\omega }_{0}t}\\\qquad\quad\;\;\,\, =(A+iS)\,{e}^{i{\omega }_{0}t}\end{array}$$

18 $${p}_{A}(t,{\omega }_{0})=(A-iS)\,{e}^{i{\omega }_{0}t}$$

**Fig. 6:**
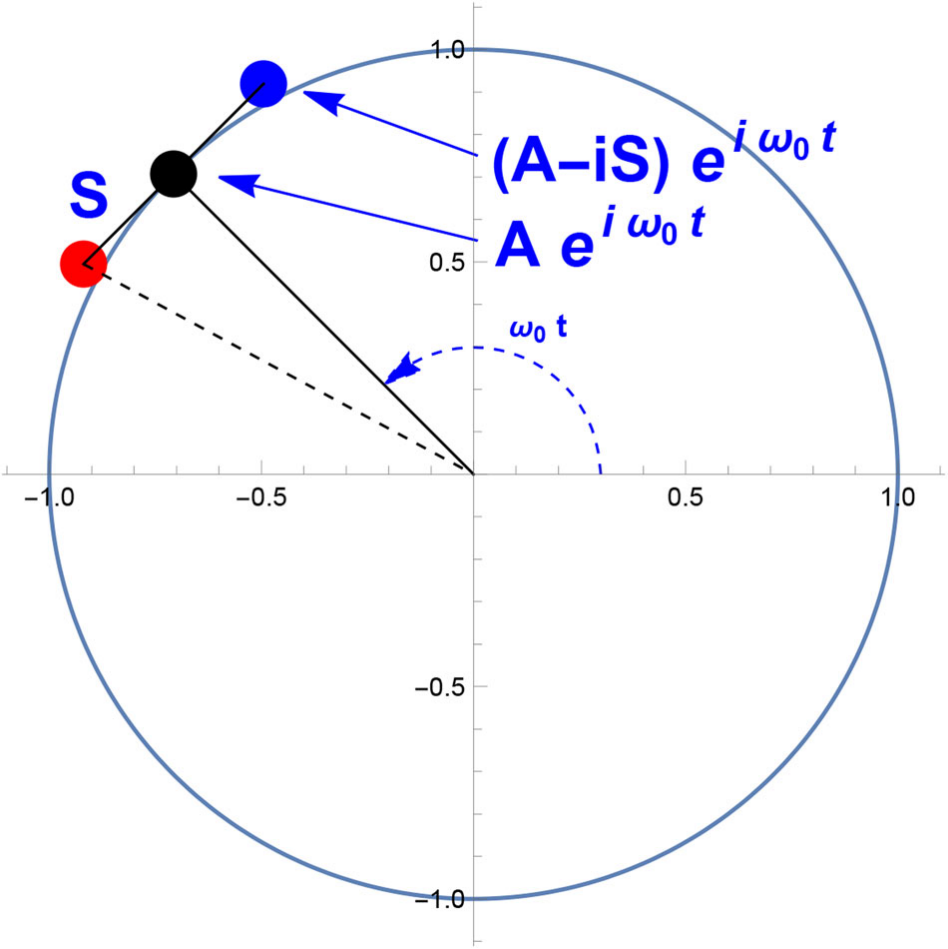
Geometric description of Point-Point Equation. This image shows the geometry of the mathematics described in Derivation of Point-Point Equation. The black point shows the location of the central axis described in Eq. (16). The red and blue points show the locations of the points described in Eqs (17) and (18). This graphic is represented in the inertial reference frame.

A plot of these locations is shown in Fig. 6. We can simplify these equations by substitution.

19 $$\begin{array}{ll}{p}_{k}(t,\omega )=(A{\pm }_{k}iS)\,{e}^{i{\omega }_{0}t}\\\qquad\quad\;\, ={\Omega }_{k}\,{e}^{i{\omega }_{0}t}\end{array}$$

where Ω_*k*_ = *A* ± _*k*_*iS*. When the location is counterclockwise from the direction of *T*_*A*_, the top symbol is used “ + .” When the location is clockwise from the direction of *T*_*A*_, the bottom symbol is used, in this case “ − .” The subscript *k* allows indexing of the locations or hands at different times or positions. For example, at time *t*_0_, the left hand might be at position *p*_+_(*t*_0_) and at a later time *p*_−_(*t*_0_ + *τ*) the right hand could be at a different position.

It may be convenient to represent Ω_*k*_ in angular rather than rectilinear coordinates and thus we may choose to use the following form instead.

20 $$\begin{array}{ll}{\Omega }_{k}=\,| \Omega | \,{e}^{{\pm }_{k}i\theta }\\\; {p}_{k}=\,| \Omega | \,{e}^{{\pm }_{k}i({\omega }_{0}t+\theta )}\end{array}$$

In the case of the rotating habitat, one may wish for the height from the floor to be included rather than the distance from the axis of rotation. Thus, ∣Ω∣ = *R*_*H*_ − *h*, where *R*_*H*_ is the distance from the floor to the axis of rotation and *h* is the height from the floor “up” to the location of the throw or catch.

The trajectories of objects may start at time *t*_0_ and travel for time *τ*. Thus they are caught at time *t*_0_ + *τ*. Let’s call the location from which the throw happens *p*_1_ and the catching location *p*_2_. In an inertial frame, the throw follows a straight line. An equation for the slope of the line can be written as …

21 $$B({t}_{0},\tau ,{\omega }_{0})={p}_{2}({t}_{0}+\tau )-{p}_{1}({t}_{0})$$

22 $$\begin{array}{ll}={\Omega }_{2}\,{e}^{i{\omega }_{0}({t}_{0}+\tau )}-{\Omega }_{1}\,{e}^{i{\omega }_{0}{t}_{0}}\\ ={e}^{i{\omega }_{0}{t}_{0}}\left({e}^{i{\omega }_{0}\tau }{\Omega }_{2}-{\Omega }_{1}\right)\end{array}$$

We can now write the equation of a line *T*_*L*_(*t*, *τ*, *ω*) which starts at point *p*_1_ at time *t*_0_, travels linearly toward *p*_2_ at time *t*_0_ + *τ*, and has the slope found in Eq. (22).

23 $$\begin{array}{ll}{T}_{L}(t,\tau ,{\omega }_{0})=\\\quad\; =\left(\frac{t}{\tau }\right)B({t}_{0},\tau ,{\omega }_{0})+{p}_{1}({t}_{0},\tau ,{\omega }_{0})\\\quad\; =\left(\frac{t}{\tau }\right)\,{e}^{i{\omega }_{0}{t}_{0}}\left({e}^{i{\omega }_{0}\tau }{\Omega }_{2}-{\Omega }_{1}\right)+{\Omega }_{1}\,{e}^{i{\omega }_{0}{t}_{0}}\\\quad\; ={e}^{i{\omega }_{0}{t}_{0}}\left[\frac{t}{\tau }\left({e}^{i{\omega }_{0}\tau }{\Omega }_{2}-{\Omega }_{1}\right)+{\Omega }_{1}\right]\end{array}$$

A subset of the trajectories described by Eq. (23) are shown in Fig. 1a with 0.2 *s* < *τ*_0_ < 0.5 *s* in increments of 0.1 *s*. The trajectories plotted have *ω*_0_*t*_0_ = *π*/2 to allow for symmetry about the imaginary axis.

We can find the trajectory of the balls in the rotating frame by multiplying Eq. (23) by a time dependent complex exponential rotating in the opposite direction to the body’s rotation. Namely,

24 $$\begin{array}{ll}T=\,{e}^{-i{\omega }_{1}t}\times {T}_{L}({t}_{0},\tau ,{\omega }_{0})\\\quad =\,{e}^{-i({\omega }_{1}t-{\omega }_{0}{t}_{0})}\left[\frac{t}{\tau }\left({e}^{i{\omega }_{0}\tau }\,{\Omega }_{2}-{\Omega }_{1}\right)+{\Omega }_{1}\right]\end{array}$$

where *ω*_1_ is the angular velocity of an observer rotating about the same axis. If the observer rotates at the same rotational velocity as the juggler or habitat, then *ω*_1_ = *ω*_0_. The trajectories can be visualized within the circle by plotting 0 < *t* < *τ*_0_. Some interesting behavior can also be found when *ω*_0_ ≠ *ω*_1_, *δω*_0_/*dt* ≠ 0, and/or *δω*_1_/*dt* ≠ 0. Each of these cases can be observed in the Space Juggler films online^30^.

### Trigonometric form of the point-point equation

This appendix offers the equations from Derivation of Point-Point Equation in trigonometric form. The objective in presenting the material in this format is to show that linear algebra is not the most ideal algebra in which to calculate or formulate equations which involve rotations. The incredible simplicity of using complex algebra, as was done in the rest of this paper, should be clear by the end of this appendix.

The equation of a point rotating around the origin at a distance *A* can be written as (compare to Eq. (16)) …

25 $$f(t,\omega )=A\left\langle cos(\omega t),sin(\omega t)\right\rangle$$

We can add the offset for the hands by addition (compare to Eq. (19)) …

26 $${p}_{k}(t,\omega )=f(t,\omega ){\mp }_{k}S\left\langle sin(\omega t),-cos(\omega t)\right\rangle$$

where the top sign in ∓ _*k*_ refers to the left hand and bottom sign refers to the right hand. Note the signs are inverted in comparison to Eq. (19).

Vector of trajectory from throw to catch (compare to Eq. (22)) …

27 $$\begin{array}{ll}B({t}_{0},{\tau }_{0},\omega )={p}_{2}({t}_{0}+\tau )-{p}_{1}({t}_{0})\\\qquad\qquad\quad =\left\langle A\right.cos(\omega {t}_{0}+\omega \tau ){\mp }_{2}Ssin(\omega {t}_{0}+\omega \tau )\\\qquad\qquad\quad -Acos(\omega t){\pm }_{1}Ssin(\omega t),\\\qquad\qquad\quad\;\; Asin(\omega {t}_{0}+\omega \tau ){\pm }_{2}Scos(\omega {t}_{0}+\omega \tau )\\\qquad\qquad\quad \left.-Asin(\omega t){\mp }_{1}Scos(\omega t)\right\rangle \end{array}$$

Linear trajectories (compare to Eq. (23)) …

28 $$\begin{array}{ll}{T}_{L}(t,{t}_{0},\tau ,\omega )=\\\quad\; =B({t}_{0},\tau ,\omega )\left(\frac{t}{{\tau }_{0}}\right)+{p}_{1}({t}_{0},\tau ,\omega )\\\quad\; =\left\langle \right.\left(Acos(\omega {t}_{0}+\omega \tau ){\mp }_{2}Ssin(\omega {t}_{0}+\omega \tau )\right.\\\quad\; \left.-Acos(\omega {t}_{0}){\pm }_{1}Ssin(\omega {t}_{0})\right)\left(\frac{t}{\tau }\right)\\\quad\; +Acos(\omega {t}_{0}){\mp }_{1}Ssin(\omega {t}_{0}),\\\quad\; \left(Asin(\omega {t}_{0}+\omega \tau ){\pm }_{2}Scos(\omega {t}_{0}+\omega \tau )\right.\\\quad\; \left.-Asin(\omega {t}_{0}){\mp }_{1}Scos(\omega {t}_{0})\right)\left(\frac{t}{\tau }\right)\\\quad\; \left.+Asin(\omega {t}_{0}){\pm }_{1}Scos(\omega {t}_{0})\right\rangle \end{array}$$

As the reader may observe, this form of the equations is much more unwieldy than that found in the exponential formulation in equation (23).

Rotating observer trajectories (compare to Eq. (24)), using the common rotation matrix requires taking the transpose of *T*_*L*_ which will be written as ${T}_{L}^{T}$. This can be computed using …

29 $$\begin{array}{ll}T=R(t,{\omega }_{1})\times {T}_{L}^{T}\\\quad =\left\langle \begin{array}{ll}cos(-{\omega }_{1}t)-sin(-{\omega }_{1}t)\\ sin(-{\omega }_{1}t)\,cos(-{\omega }_{1}t)\end{array}\right\rangle \times {T}_{L}^{T}\\\quad =\left\langle \begin{array}{ll}cos({\omega }_{1}t)\,sin({\omega }_{1}t)\\ -sin({\omega }_{1}t)\,cos({\omega }_{1}t)\end{array}\right\rangle \times {T}_{L}^{T}\end{array}$$

It is hopefully obvious that by looking at the previous couple of equations and comparing them to the solutions offered in Derivation of Point-Point Equation, that complex algebra is better suited for this type of work than linear algebra.

### Reporting summary

Further information on research design is available in the Nature Research Reporting Summary linked to this article.

### Supplementary information

Reporting Summary

Supplemental Material

Roulette creation animation

Rotating reference frame tracked data

Rotating space habitat in inertial and rotating frames

Experimental observations in inertial and rotating frames

## Data Availability

The data analyzed in this paper is video recorded using the microgravity analog apparatus described in the Analog Microgravity Environment section. The reader may find the videos used to create Fig. 3, the spreadsheets used for calculation and analysis to create Table 1, and Mathematica visualization code on The Open Science Framework (OSF) online repository at https://osf.io/a2sb6/. Please contact the author if you have questions about the data or techniques to repeat the experimental setup.

## Code availability

The code used in this study is available on the Open Science Framework (OSF) platform at https://osf.io/a2sb6/. The code can be accessed directly from the OSF platform.
