# Supplementary material for: Simplified equations for object trajectories in rotating space habitats and space juggling
Source: NPJ Microgravity. 2023 Oct 12;9:82. doi: 10.1038/s41526-023-00328-6 (PMC10570307; doi:10.1038/s41526-023-00328-6)
Supplement: Supplementary file 1 — Reporting Summary [file 41526_2023_328_MOESM1_ESM.pdf]

nature portfolio

Corresponding author(s): R. Adam Dipert

Last updated by author(s): Apr 7, 2023

Reporting Summary

Nature Portfolio wishes to improve the reproducibility of the work that we publish. This form provides structure for consistency and transparency in reporting. For further information on Nature Portfolio policies, see our Editorial Policies and the Editorial Policy Checklist.

Statistics

For all statistical analyses, confirm that the following items are present in the figure legend, table legend, main text, or Methods section.

n/a	Confirmed	
☐	☒	The exact sample size (*n*) for each experimental group/condition, given as a discrete number and unit of measurement
☐	☒	A statement on whether measurements were taken from distinct samples or whether the same sample was measured repeatedly
☐	☒	The statistical test(s) used AND whether they are one- or two-sided<br>*Only common tests should be described solely by name; describe more complex techniques in the Methods section.*
☐	☒	A description of all covariates tested
☐	☒	A description of any assumptions or corrections, such as tests of normality and adjustment for multiple comparisons
☐	☒	A full description of the statistical parameters including central tendency (e.g. means) or other basic estimates (e.g. regression coefficient) AND variation (e.g. standard deviation) or associated estimates of uncertainty (e.g. confidence intervals)
☐	☒	For null hypothesis testing, the test statistic (e.g. *F*, *t*, *r*) with confidence intervals, effect sizes, degrees of freedom and *P* value noted<br>*Give P values as exact values whenever suitable.*
☐	☒	For Bayesian analysis, information on the choice of priors and Markov chain Monte Carlo settings
☐	☒	For hierarchical and complex designs, identification of the appropriate level for tests and full reporting of outcomes
☐	☒	Estimates of effect sizes (e.g. Cohen's *d*, Pearson's *r*), indicating how they were calculated

Our web collection on statistics for biologists contains articles on many of the points above.

Software and code

Policy information about availability of computer code

Data collection	Data was collected using a video camera.
Data analysis	Analysis was conducted on Microsoft Excel, Python, and Mathematica. The Excel spreadsheets and Mathematica code are available on the OSF platform.

For manuscripts utilizing custom algorithms or software that are central to the research but not yet described in published literature, software must be made available to editors and reviewers. We strongly encourage code deposition in a community repository (e.g. GitHub). See the Nature Portfolio guidelines for submitting code & software for further information.

Data

Policy information about availability of data

All manuscripts must include a data availability statement. This statement should provide the following information, where applicable:

- Accession codes, unique identifiers, or web links for publicly available datasets
- A description of any restrictions on data availability
- For clinical datasets or third party data, please ensure that the statement adheres to our policy

The data analyzed in this paper is video recorded using the microgravity analog apparatus described in the Analog Microgravity Environment section. The reader may find the videos used to create figures 3, the spreadsheets used for calculation and analysis to create table 1, and Mathematica visualization code on The Open

Science Framework (OSF) online repository at https://osf.io/a2sb6/. Please contact the author if you have questions about the data or techniques to repeat the experimental set up.

Human research participants

Policy information about studies involving human research participants and Sex and Gender in Research.

Reporting on sex and gender	Sex and gender data was not collected for this study.
Population characteristics	The data used for this study was deemed "Secondary data" by the IRB at NC State University. The original purpose of collecting the data was not to perform research. Therefore, no particular population characteristics were collected.
Recruitment	"Secondary data" (see above)
Ethics oversight	IRB at North Carolina State University, protocol number 37225523

Note that full information on the approval of the study protocol must also be provided in the manuscript.

Field-specific reporting

Please select the one below that is the best fit for your research. If you are not sure, read the appropriate sections before making your selection.

☒ Life sciences ☐ Behavioural & social sciences ☐ Ecological, evolutionary & environmental sciences

For a reference copy of the document with all sections, see nature.com/documents/nr-reporting-summary-flat.pdf

Life sciences study design

All studies must disclose on these points even when the disclosure is negative.

Sample size	The data used in this study is the tracking of balls which were thrown by the author. The sample size is the number of frames of video collected during a particular trajectory. Sample sizes are on the order of 60-86 points.
Data exclusions	No data was excluded.
Replication	Tests were repeated several hundred times to identify if the trajectories were consistent with the mathematical theory. In the article, only a small number of trajectories are fit and shown to allow clear visual confirmation of the fit.
Randomization	As secondary data was used (see above), no randomization was included.
Blinding	As secondary data was used (see above), no blinding was included in this study.

Reporting for specific materials, systems and methods

We require information from authors about some types of materials, experimental systems and methods used in many studies. Here, indicate whether each material, system or method listed is relevant to your study. If you are not sure if a list item applies to your research, read the appropriate section before selecting a response.

Materials & experimental systems

n/a	Involved in the study	
☒	☐	Antibodies
☒	☐	Eukaryotic cell lines
☒	☐	Palaeontology and archaeology
☒	☐	Animals and other organisms
☒	☐	Clinical data
☒	☐	Dual use research of concern

Methods

n/a	Involved in the study	
☒	☐	ChIP-seq
☒	☐	Flow cytometry
☒	☐	MRI-based neuroimaging
